# Impact of aging on cardiovascular dynamics and heart rate variability during passive head‐up tilt

**DOI:** 10.14814/phy2.70477

**Published:** 2025-07-20

**Authors:** Manoj Kumar Choudhary, Kati Holmström, Heidi Bouquin, Tuomas P. Saarinen, Jenni K. Koskela, Essi Pietilä, Lauri Suojanen, Jukka T. Mustonen, Pasi I. Nevalainen, Ilkka H. Pörsti

**Affiliations:** ^1^ Faculty of Medicine and Health Technology Tampere University Tampere Finland; ^2^ Department of Internal Medicine Hospital Nova Jyväskylä Finland; ^3^ Department of Internal Medicine Tampere University Hospital Tampere Finland; ^4^ Finnish Cardiovascular Research Centre Tampere Tampere University Tampere Finland

**Keywords:** aging, blood pressure, head‐up tilt, heart rate, hemodynamics

## Abstract

During aging, arterial stiffness and blood pressure (BP) increase, while heart rate variability (HRV) decreases. We examined age‐related hemodynamics in 522 individuals without cardiovascular disease during passive head‐up tilt (HUT) using pulse wave analysis, whole‐body impedance cardiography, and HRV analysis. Mean age was 44 years, body mass index 26 kg/m^2^, and office BP 138/88 mmHg. Cardiac output was 7% higher in the youngest quartile than the oldest. With increasing age, aortic BP, pulse pressure, pulse wave velocity, wave reflection, and systemic vascular resistance increased, while heart rate, pulse pressure amplification, and aortic reflection time decreased. During HUT, aging was linked to greater reductions in systolic BP, pulse pressure, and stroke volume, as well as diminished heart rate increase and reduced aortic reflection time shortening. HRV in low‐frequency (LF) and high‐frequency (HF) power decreased with age. However, the supine LF:HF ratio was higher in the oldest quartile than the youngest, a pattern not seen during HUT. In conclusion, higher arterial stiffness and systemic vascular resistance likely contribute to age‐related rises in BP and pulse pressure, reduced pulse pressure amplification, and shorter aortic reflection time. Although HRV decreased with age, the supine LF:HF ratio increased, a change absent in the upright position.

## 1 INTRODUCTION

Aging is accompanied by progressive increases in systolic blood pressure (BP) and pulse pressure, both of which are well‐established independent risk factors for cardiovascular morbidity and mortality (Franklin et al., 1997; Laurent et al., 2006; Laurent & Boutouyrie, 2020; Mitchell et al., 2007). These changes reflect underlying pathological processes, including arterial stiffening, impaired vascular compliance, and endothelial dysfunction (Laurent et al., 2006, 2019; Laurent & Boutouyrie, 2020). Elevated systolic BP and widened pulse pressure contribute to increased left ventricular afterload, reduced coronary perfusion, and vascular damage, thereby accelerating the development of left ventricular hypertrophy, atherosclerosis, and heart failure (Blacher et al., 1979; Franklin et al., 1999; Wilkinson et al., 2001). Understanding the mechanisms that drive these hemodynamic changes is essential for identifying early markers of cardiovascular risk and potential targets for prevention, particularly as global populations continue to age.

The age‐related increase in BP is largely driven by progressive stiffening of large elastic arteries, which enhances pulse wave velocity (PWV) and wave reflection, leading to elevated systolic pressure and pulse pressure (Kaess et al., 2012; Laurent et al., 2006; Laurent & Boutouyrie, 2020). Arterial stiffness progressively increases with age, representing an arterial pathology that disrupts vascular wave dynamics (Laurent et al., 2006; Laurent & Boutouyrie, 2020; Mitchell et al., 2007) and may precede the development of hypertension (Kaess et al., 2012; Laurent & Boutouyrie, 2020). Higher aortic stiffness has been shown to elevate the risk of cardiovascular events (Mitchell et al., 2010). Data from the Framingham Heart Study revealed that the prevalence of high‐risk aortic stiffness was approximately 1% in both sexes before the age of 50 but rose dramatically to over 64% in men and 74% in women aged ≥70 years (Mitchell et al., 2007).

Cardiovascular status assessments typically involve recording BP and heart rate in the seated position (Stergiou et al., 2021). However, BP and heart rate measurements in the standing position can provide relevant clinical information, particularly in elderly individuals, patients with neurodegenerative diseases or diabetes, or when postural hypotension is suspected (Stergiou et al., 2021). Even in the absence of cardiovascular disease, systolic BP responses to orthostatic challenges can vary widely (Suojanen et al., 2021). Although a significant portion of daily activities occurs in the upright position, detailed information on upright hemodynamics remains limited, but such information could provide relevant information for cardiovascular risk evaluation (Palatini, 2023; Palatini et al., 2022). When standing, blood pools in the lower extremities, reducing venous return and cardiac output (Suojanen et al., 2021; Wieling et al., 2022). To counteract this, sympathetic tone increases while parasympathetic tone decreases, leading to elevated heart rate and systemic vascular resistance (SVR) to maintain BP (Avolio & Parati, 2011; Reulecke et al., 2016; Tikkakoski et al., 2013). However, individual differences exist in phenotypic responses, particularly in the extent of changes in SVR and cardiac output during upright posture (Tahvanainen et al., 2016).

Aging is associated with alterations in cardiovascular autonomic regulation, both at rest and during physical challenges. Several studies have observed that in response to head‐up tilt (HUT), elderly individuals exhibit lower increases in heart rate compared to younger individuals (Fagard et al., 1994; Laitinen et al., 2004; Singam et al., 2019; Smith et al., 1987). This attenuated response has been attributed to age‐related reductions in baroreceptor sensitivity (Laitinen et al., 2004; Paleczny et al., 2014). During passive HUT, aging is also linked to reduced heart rate variability (HRV), a measure that can be used to evaluate the functional age of the heart (Colosimo et al., 1997).

Although age‐related changes in hemodynamics have been investigated in numerous studies, information about the influence of aging on cardiovascular regulation during upright posture remains incomplete. Here, we tested the hypothesis whether age‐related changes in the hemodynamic variables are accentuated during physical challenge induced by upright posture, and investigated the effects of aging on central hemodynamics, wave reflection, SVR, cardiac function, and autonomic modulation of heart rate during passive HUT. The present study included 522 individuals without cardiovascular disease, none of whom used medications with direct cardiovascular effects, allowing for the assessment of normative age‐related cardiovascular responses.

## 2 MATERIALS AND METHODS

### 2.1 Study population

This investigation is part of the DYNAMIC study (EudraCT 2006‐002065‐39, Clinical Trials registration NCT01742702), with a focus on non‐invasive hemodynamics. Approval for the study was obtained from the Ethics Committee of Tampere University Hospital (study code R06086M), adhering to the principles outlined in the Declaration of Helsinki. Participants were enrolled sequentially based on the receipt of their contact information by research nurses. All participants provided written informed consent. The participant recruitment has been previously published (Bouquin et al., 2024), and altogether 522 subjects from all 1442 studied were included. The study's exclusion criteria included individuals with a medical history of (1) coronary artery disease, (2) stroke, (3) heart failure, (4) valvular heart disease, (5) diabetes, (6) chronic kidney disease, (7) secondary hypertension, (8) alcohol or substance abuse, (9) psychiatric illnesses with the exception of mild depression or anxiety, (10) heart rhythm other than sinus rhythm, and (11) use of medications with direct influences on cardiovascular function.

A medical doctor performed physical examinations and office BP measurements. Routine laboratory analyses for elevated BP were conducted in all enrolled participants, adhering to the guidelines outlined by the European Society of Hypertension (Stergiou et al., 2021). Along with medical history, documentation included lifestyle habits, dietary supplement usage, medications, and other substances not classified as drugs and the number of ≥30‐min bouts of exercise per week. Smoking habits and alcohol consumption were recorded, with alcohol intake quantified in standard drinks (approximately 12 g of absolute alcohol) per week. To familiarize participants with the head‐up tilt (HUT) procedure and to ensure the absence of autonomic dysfunction, all participants first underwent an introductory HUT test. None of the individuals included in the study exhibited abnormal autonomic responses, such as orthostatic hypotension or signs of excessive postural tachycardia.

The study included 273 men and 249 women, aged 19–72 years, who were either normotensive or had untreated primary hypertension (Table 1). Histograms showing the distribution of supine aortic systolic/diastolic blood pressure and heart rate are presented in Figures S1 and S2, illustrating a wide range of values across all age groups. In total, 319 participants (61.1%) reported using some form of medication or supplement (Supplemental Table—Data S1): female hormones (estrogen, progestin or their combination, *n* = 76), vitamin D and other dietary supplements (vitamins, minerals, omega‐3 fatty acids) (*n* = 95), antidepressants (*n* = 32), hormonal intrauterine devices (IUDs) (*n* = 23), thyroxine (*n* = 15), statins (*n* = 13), ezetimibe (*n* = 1), inhaled glucocorticoids (*n* = 15), antihistamines (*n* = 16), proton pump inhibitors (*n* = 13), low dose acetylsalicylic acid (*n* = 6), non‐steroidal anti‐inflammatory drugs (*n* = 5), topically applied estrogens (*n* = 6), inhaled beta2‐mimetics (*n* = 7), allopurinol (*n* = 2), warfarin (*n* = 1).

**TABLE 1:** Demographics, clinical characteristics, and cardiac ejection kinetics in the different age groups.

Variable	Overall	Age group (rounded to the nearest full decade)			
		30 (*n* = 128)	40 (*n* = 135)	50 (*n* = 133)	60 (*n* = 126)
Males/females (%)	273/249	68/60	71/64	67/66	67/59
Age (years)	44.1 (11.7)	28.1 (4.4)	40.6 (3.1)^*^	49.5 (2.6)*, ^†^	58.7 (4.0)*, ^†^, ^‡^
Weight (kg)	79.3 (15.4)	74.6 (14.6)	80.8 (15.9)*	81.2 (17.3)*	80.5 (12.3)*
Height (cm)	172.9 (9.2)	173.7 (9.7)	173.5 (9.0)	172.5 (9.3)	171.9 (8.7)
Body mass index (kg/m^2^)	26.4 (4.3)	24.6 (3.9)	26.7 (4.2)*	27.1 (4.7)*	27.2 (3.6)*
Current smokers (number/percentage)	73/14	19/14.8	15/11.1	23/17.3	16/12.7
Use of alcohol (standard drinks/week)	3 [1–5]	3 [1–5]	3 [1–6]	2 [1–5]	3 [1–6]
Seated office measurements					
Heart rate (beats/min)	67 (10)	68 (10)	65 (9)*	68 (12)^†^	65 (8)*, ^‡^
Systolic blood pressure (mmHg)	138 (19)	130 (15)	133 (17)*	141 (20)*, ^†^	148 (20)*, ^†^, ^‡^
Diastolic blood pressure (mmHg)	88 (12)	84 (13)	88 (12)*	91 (12)*, ^†^	91 (9)*, ^†^
Cardiac ejection kinetics					
Ejection duration, supine (ms)	329 (19)	329 (19)	328 (17)	327 (19)	332 (21)
Ejection duration, head‐up tilt (ms)	269 (22)	259 (21)	268 (21)*	273 (21)*	277 (21)*, ^†^
Ejection shortening during head‐up tilt (ms)	−60 (18)	−69 (17)	−61 (19)*	−54 (15)*, ^†^	−54 (15)*, ^†^

*Note*: Results shown as mean (standard deviation), median [25th–75th percentile], or number/percentage of participants.

* *p <* 0.05 versus group 30.

^†^ *p <* 0.05 versus group 40.

^‡^ *p <* 0.05 versus group 50.

### 2.2 Instrumentation

A single‐channel electrocardiogram (ECG) and changes in body electrical impedance were monitored using whole‐body impedance cardiography (CircMon®, JR Medical Ltd., Tallinn, Estonia) (Kangas et al., 2016; Kööbi et al., 1997, 2003; Tahvanainen, Koskela, et al., 2009). The electrode configuration of the CircMon® device has been described before (Tahvanainen, Koskela, et al., 2009). Briefly, two electrodes were placed on the distal parts of the limbs, proximal to the wrists and ankles, with their centers approximately 5 cm apart; voltage electrodes were positioned proximally to the current electrodes. Distal impedance was also recorded from the popliteal artery region, with the active electrode placed laterally at the knee and the reference electrode on the calf, approximately 20 cm apart. ECG was recorded at 200 Hz for heart rate and HRV analysis (Bouquin et al., 2024).

Pulse wave analysis was conducted using a signal from a tonometric sensor (Colin BP‐508 T, Colin Medical Instruments Corp., USA) positioned over the radial artery of the left wrist, with oscillometric BP calibration from the right upper arm approximately every 2.5 min (Kangas et al., 2016; Tahvanainen, Koskela, et al., 2009). The left arm was supported at a 90° angle to maintain alignment with heart level in both supine and upright positions.

### 2.3 Experimental protocol

**FIGURE 1:**
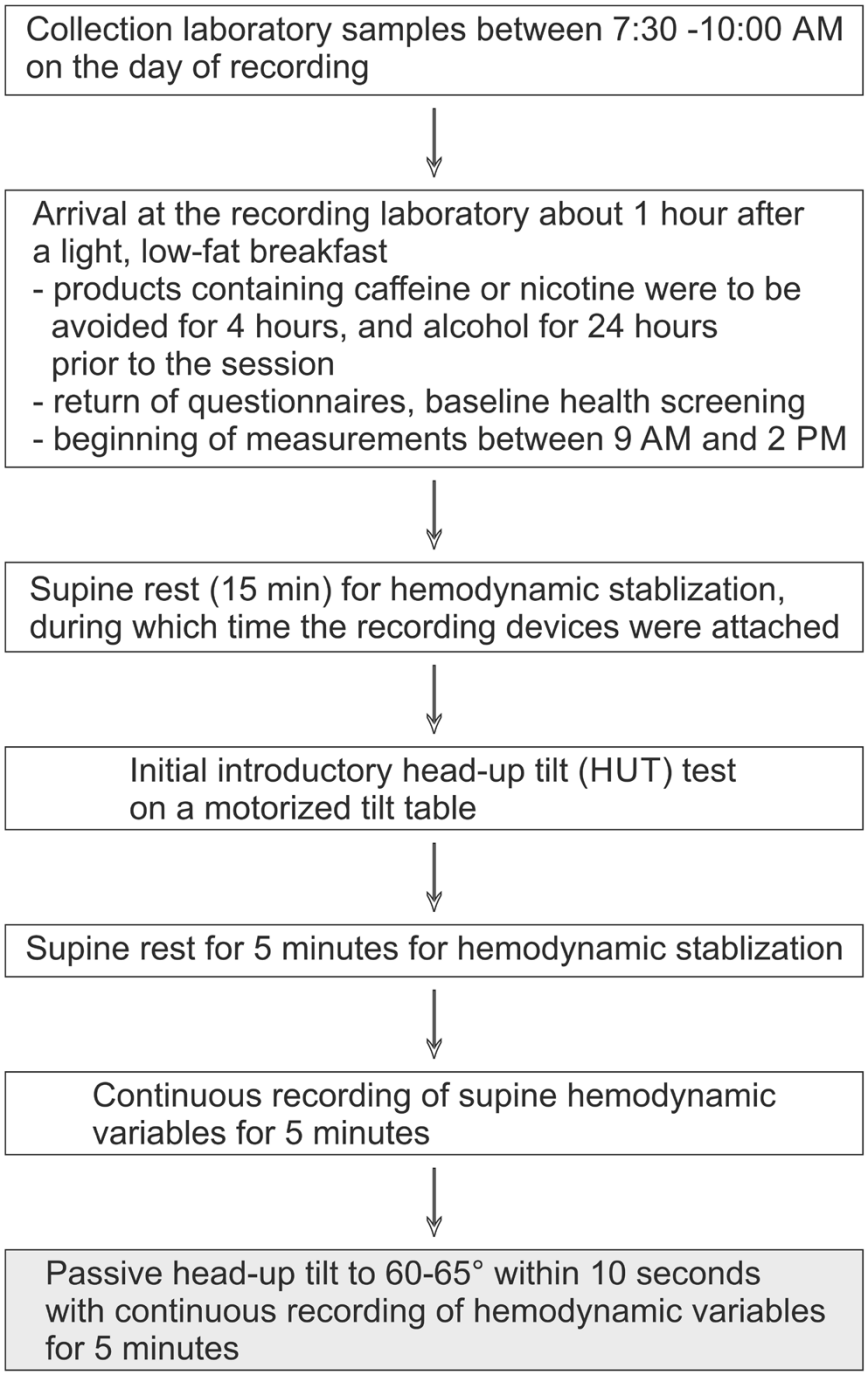
Instrumentation and experimental protocol flowchart.

Hemodynamic recordings were performed by a trained research nurse in a quiet, temperature‐controlled laboratory following established protocols (Bouquin et al., 2024; Kangas et al., 2016; Tahvanainen, Koskela, et al., 2009) (Figure 1). Participants fasted overnight, with laboratory samples collected between 7:30 and 10:00 am. Measurements began between 9:00 am and 2:00 pm to account for circadian variation. A light low‐fat breakfast (e.g., juice or yoghurt) or light lunch (e.g., salad) was allowed, while caffeine, nicotine, vigorous physical activity within 4 h, and alcohol consumption within 24 h prior to recordings were prohibited.

Approximately 1 h after light food intake, participants arrived at the laboratory where anthropometric data (height, weight, waist, and hip circumference) were recorded, and study questionnaires were reviewed. Participants then rested supine for 15 min while devices were attached, followed by an introductory HUT for familiarization and calibration.

Following the introductory tilt, participants rested supine for an additional 5 min to allow stabilization of hemodynamic parameters. Hemodynamic recordings were then performed in the supine position for 5 min. Subsequently, participants were passively tilted to a 60°–65° upright position using a motorized tilt table with foot support, with the tilt maneuver completed in approximately 10 seconds. From the beginning of the HUT, data was recorded continuously for 5 min (Figure 1). After HUT, mean arterial pressure and heart rate have been reported to reach a steady‐state within less than 2.5 min (Heldt et al., 2002, 2003).

### 2.4 Pulse wave analysis and whole‐body impedance cardiography

The tonometric radial artery pulse waveform was transmitted to the SphygmoCor PWMx system (AtCor Medical, New South Wales, Australia) to provide continuous estimates of aortic BP using a validated generalized transfer function (Chen et al., 1997) (Figure 1). This method has been approved by the US Food and Drug Administration (K070795). Beat‐to‐beat heart rate, stroke volume, and cardiac output were assessed from the body electrical signal by the CircMon® software (Kangas et al., 2016; Kööbi et al., 1997, 2003). When the pressure wave enters the aortic arch, whole‐body impedance decreases, as detected by voltage electrodes placed on the distal extremities. The CircMon® device calculates stroke volume using demographic data and changes in body impedance, and determines PWV from the time difference between the onset of impedance decrease in the whole‐body signal and the popliteal region signal. PWV measured using whole‐body impedance cardiography corresponds well to values recorded using the tonometric SphygmoCor method or ultrasound (Kööbi et al., 2003; Wilenius et al., 2016).

SVR and left cardiac work were determined from the tonometric BP and cardiac output evaluated using CircMon® (Kangas et al., 2016). Stroke volume, cardiac output, SVR, and left cardiac work were adjusted to body surface area and presented as indexes (SI, CI, SVR index, and LCWI, respectively). LCWI was calculated using the formula: 0.0143 × (mean aortic pressure − pulmonary artery occlusion pressure) × CI (Gorlin et al., 1955; Kangas et al., 2016). Assumed mean normal values of central venous pressure (3 mmHg) and pulmonary arterial occlusion pressure (6 mmHg) were utilized in the calculations (Kangas et al., 2016; Kööbi et al., 1997, 2003). Peripheral pulse pressure was defined as the difference between brachial systolic and diastolic BPs, and central pulse pressure as the difference between aortic systolic and diastolic pressures. Pulse pressure amplification was calculated as the ratio of radial pulse pressure to central pulse pressure (Suonsyrjä et al., 2024; Tahvanainen, Leskinen, et al., 2009).

### 2.5 Heart rate variability analyses

HRV was evaluated from the RR intervals of the single‐channel electrocardiogram (ECG) recordings obtained using the CircMon® system (sampling rate: 200 Hz). Normal R–R intervals were identified, and ectopic beats were defined as intervals with a variation exceeding 20% from the preceding beat. Artifacts and ectopic beats were corrected using cubic spline interpolation. Frequency‐domain HRV analysis was performed using MATLAB software (MathWorks Inc., Natick, MA, USA). Power spectral density was computed via Fast Fourier Transformation. The following spectral components were analyzed: low‐frequency (LF) power (0.04–0.15 Hz), high‐frequency (HF) power (0.15–0.40 Hz), and the LF/HF ratio as an index reflecting cardiac sympathovagal balance (Task force of the European Society of Cardiology the north American Society of Pacing Electrophysiology, 1996).

### 2.6 Laboratory analyses

Blood and urine samples were collected following approximately 12 h of fasting. The Quantitative Insulin Sensitivity Check Index (QUICKI) was calculated based on fasting plasma glucose and insulin levels (Katz et al., 2000). Plasma concentrations of C‐reactive protein (CRP), sodium, potassium, calcium, phosphate, glucose, creatinine, triglycerides, and total, high‐density, and low‐density lipoprotein cholesterol were determined using the Cobas Integra 700/800 chemistry analyzer (Roche Diagnostics, Basel, Switzerland). Blood cell counts were analyzed using ADVIA 2120 hematology systems (Siemens, Munich, Germany). Concentrations of parathyroid hormone (PTH) and insulin were measured using electrochemiluminescence immunoassay (Cobas e411, Roche Diagnostics, Basel, Switzerland, catalogue numbers 11972103160 and 12017547122), and calcidiol (25(OH)D_3_) concentrations using enzyme immunoassay (Immunodiagnostic Systems, Boldon, UK, product code AC‐57SF1). Estimated GFR (eGFR) was calculated using the combined creatinine and cystatin C formula (Inker et al., 2012).

## 3 STATISTICS

Continuous variables were expressed as mean, standard error of the mean, or standard deviation (SD), or as median [25th–75th percentile], as appropriate. Baseline characteristics and laboratory values were presented as quartiles of age rounded to the nearest full decade (Tables 1 and 2). Demographic and laboratory data were analyzed using the analysis of variance (ANOVA) or the Kruskal‐Wallis test, as appropriate. The homogeneity of variances was tested with Levene's test, and Pearson's (r_P_) or Spearman's correlations (r_S_) were computed. The HRV statistics were analyzed from logarithmically transformed values recorded during the 5‐min periods in supine and upright postures. Due to the heart rate dependency of HRV, these analyses were also adjusted to the mean supine and upright RR intervals (Sacha, 2013).

**TABLE 2:** Laboratory results of blood and plasma samples in quartiles across the different age groups.

Laboratory results	Overall	Age group (rounded to the nearest full decade)			
		30 (*n* = 128)	40 (*n* = 135)	50 (*n* = 133)	60 (*n* = 126)
Blood hemoglobin (g/L)	144 (12)	143 (12)	144 (13)	144 (12)	144 (10)
Potassium (mmol/L)	3.8 (0.3)	3.8 (0.3)	3.8 (0.3)	3.8 (0.3)	3.8 (0.2)
Sodium (mmol/L)	140.4 (2)	140.5 (2)	139.9 (2)	140.4 (2)	140.9 (2)^†^
Parathyroid hormone (pmol/L)	4.6 (1.8)	4.2 (1.9)	4.5 (2.0)	4.7 (1.7)	4.8 (1.7)*
Calcium (mmol/L)	2.3 (0.1)	2.3 (0.1)	2.3 (0.1)	2.3 (0.1)	2.3 (0.1)
25 (OH) D_3_ (nmol/L)	73 (372)	74 (44)	72 (33)	70 (39)	76 (32)
Creatinine (μmol/L)	74 (14)	75 (14)	75 (14)	73 (14)	73 (13)
Cystatin C (mg/L)	0.84 (0.15)	0.78 (0.1)	0.81 (0.1)	0.86 (0.2)*, ^†^	0.90 (0.1)*, ^†^
eGFR (ml/min/1.73 m^2^)^#^	99 (15)	110 (14)	102 (12)*	95 (12)*, ^†^	88 (12)*, ^†^, ^‡^
Total cholesterol (mmol/L)	5.1 (1.0)	4.5 (0.9)	5.0 (0.9)*	5.4 (1.0)*, ^†^	5.6 (1.0)*, ^†^
HDL cholesterol (mmol/L)	1.6 (0.4)	1.6 (0.4)	1.6 (0.5)	1.5 (0.4)	1.7 (0.4)^‡^
LDL cholesterol (mmol/L)	3.0 (1.0)	2.5 (0.9)	2.9 (0.8)*	3.3 (1.0)*, ^†^	3.4 (0.9)*, ^†^
Triglycerides (mmol/L)	0.99 [0.7–1.4]	0.86 [0.6–1.2]	1.00 [0.7–1.6]*	1.12 [0.8–1.6]*	0.98 [0.9–1.4]
C‐reactive protein (mg/L)	0.7 [0.5–1.7]	0.5 [0.5–1.1]	0.6 [0.5–1.9]	0.8 [0.5–1.5]	1.1 [0.5–2.2]*
Fasting glucose (mmol/L)	5.4 (0.6)	5.2 (0.4)	5.4 (0.5)*	5.5 (0.6)*	5.6 (0.7)*, ^†^
Insulin (mU/L)	8.3 (5.8)	8.0 (5.2)	8.6 (5.8)	8.8 (7.0)	7.7 (5.1)
QUICKI	0.358 (0.044)	0.361 (0.041)	0.356 (0.037)	0.357 (0.041)	0.361 (0.054)

*Note*: Mean (standard deviation) or median [25th–75th percentile]; eGFR derived from creatinine and cystatin C (Inker et al., 2012).

Abbreviations: eGFR, estimated glomerular filtration rate; HDL, high density lipoprotein; LDL, low density lipoprotein; QUICKI, quantitative insulin sensitivity check index.

* *p <* 0.05 versus group 30.

^†^ *p <* 0.05 versus group 40.

^‡^ *p <* 0.05 versus group 50.

To assess the hemodynamic differences across age quartiles during the 5‐min supine and upright recordings, a generalized estimating equations (GEE) model was applied. The autoregressive correlation matrix option was applied to account for autocorrelation between successive hemodynamic recordings. Statistical adjustments were made for differences in age, sex, BMI, plasma lipids, and cystatin C concentration by including these variables as covariates in the analyses. For PWV analyses, additional adjustments were made for mean aortic BP following the guidelines (Townsend et al., 2015). The Bonferroni correction was employed for all post‐hoc analyses, with *p* < 0.05 considered statistically significant. Statistical analyses were conducted using SPSS version 29.0 (IBM SPSS Statistics, Armonk, NY, USA).

## 4 RESULTS

### 4.1 Study population, results of blood and plasma analyses

The analysis included 522 subjects, comprising 273 males (52%) and 249 females (48%), divided into sex‐specific quartiles of age groups. The groups were named by rounding the mean ages to the nearest decade (Table 1). Participant age ranged from 19 to 72 years, while mean (SD) age was 44.1 (11.7) years, mean BMI was 26.4 (4.3) kg/m^2^, prevalence of smokers 14%, mean office systolic/diastolic BP 138/88 (19/12) mmHg, and mean office heart rate was 67 (10) beats per minute. The age group 30 showed the lowest values for weight, BMI, and office BP. Office BPs showed a clear age‐dependent increase, with the exception of similar diastolic BPs in the age groups 50 and 60 (Table 1).

There were no significant differences in the prevalence of medication or dietary supplement use between the study groups, with the exception of a higher prevalence of hormonal IUD use in the 40‐year age group compared to the 30‐year group (*p* = 0.012), and a higher prevalence of statin use in the 50‐year group compared to the 40‐year group (*p* = 0.024) (Supplemental Table—Data S1).

Blood hemoglobin and plasma concentrations of potassium, calcium, 25(OH)D_3_, creatinine, and insulin, as well as insulin sensitivity assessed using the quantitative insulin sensitivity check index (QUICKI), were similar across all age groups (Table 2). Plasma sodium levels were slightly higher in age group 60 compared to age group 40, while PTH and CRP concentrations showed minor elevations in age group 60 compared to age group 30.

Cystatin C levels were higher in age groups 50 and 60 than in the age groups 30 and 40, whereas eGFR declined with age, reaching the lowest values in age group 60. Total and LDL cholesterol were lowest in age group 30 and highest in age groups 50 and 60. HDL cholesterol was higher in age group 60 compared to age group 50. Fasting plasma glucose was lowest in age group 30, while glucose concentrations were slightly higher in age group 60 than in age group 40 (Table 2).

### 4.2 Hemodynamic analyses

**FIGURE 2:**
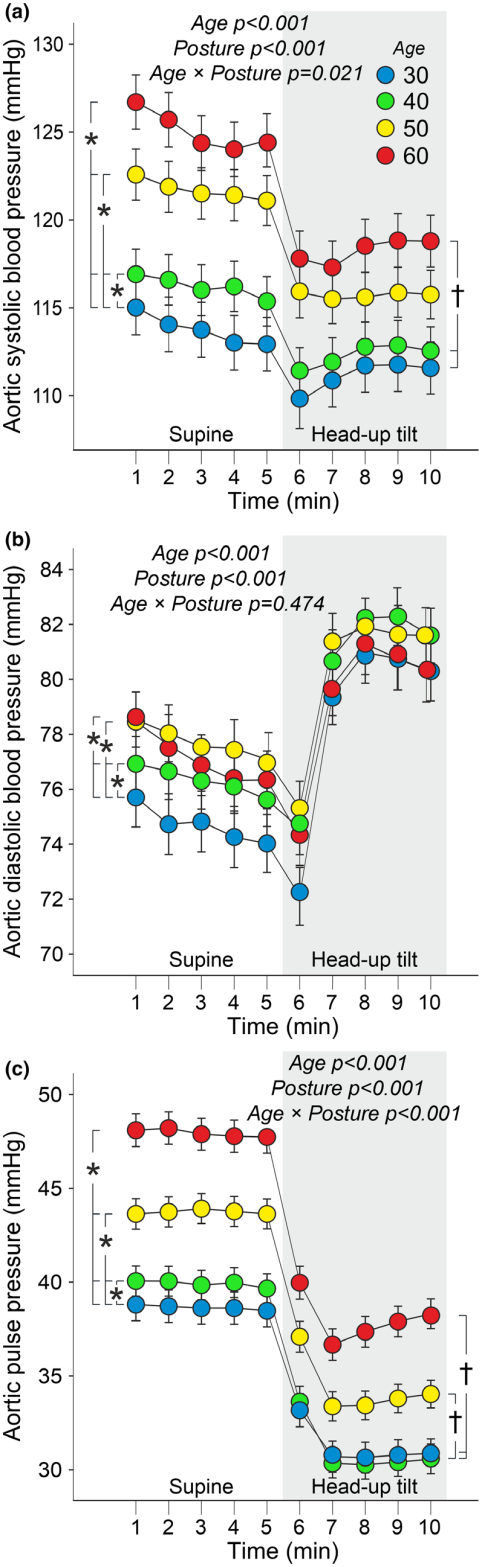
Line graphs illustrate aortic systolic (a) and diastolic (b) blood pressure and aortic pulse pressure (c) during supine position and passive head‐up tilt in different age groups. The statistical analyses were performed using generalized estimating equations and were adjusted for sex, body mass index, plasma lipids, and cystatin C; data are presented as mean ± standard error of the mean to illustrate the precision of group means rather than within‐group dispersion; *p < 0.05 between age groups, †*p* < 0.05 for the interaction between age and posture.

Mean aortic systolic BP and pulse pressure gradually increased with age. During the HUT, a significant posture interaction was observed: individuals in their 60s experienced a greater decrease in systolic BP compared to the two youngest age groups. Additionally, the decrease in aortic pulse pressure during HUT was more pronounced in the two oldest age groups compared to the two youngest (Figure 2a,c).

Differences in aortic diastolic BP were small, but average values were highest in age groups 50 and 60 and were also higher in age group 40 compared to age group 30. Aortic diastolic BP increased similarly across all age groups during HUT (Figure 2b).

**FIGURE 3:**
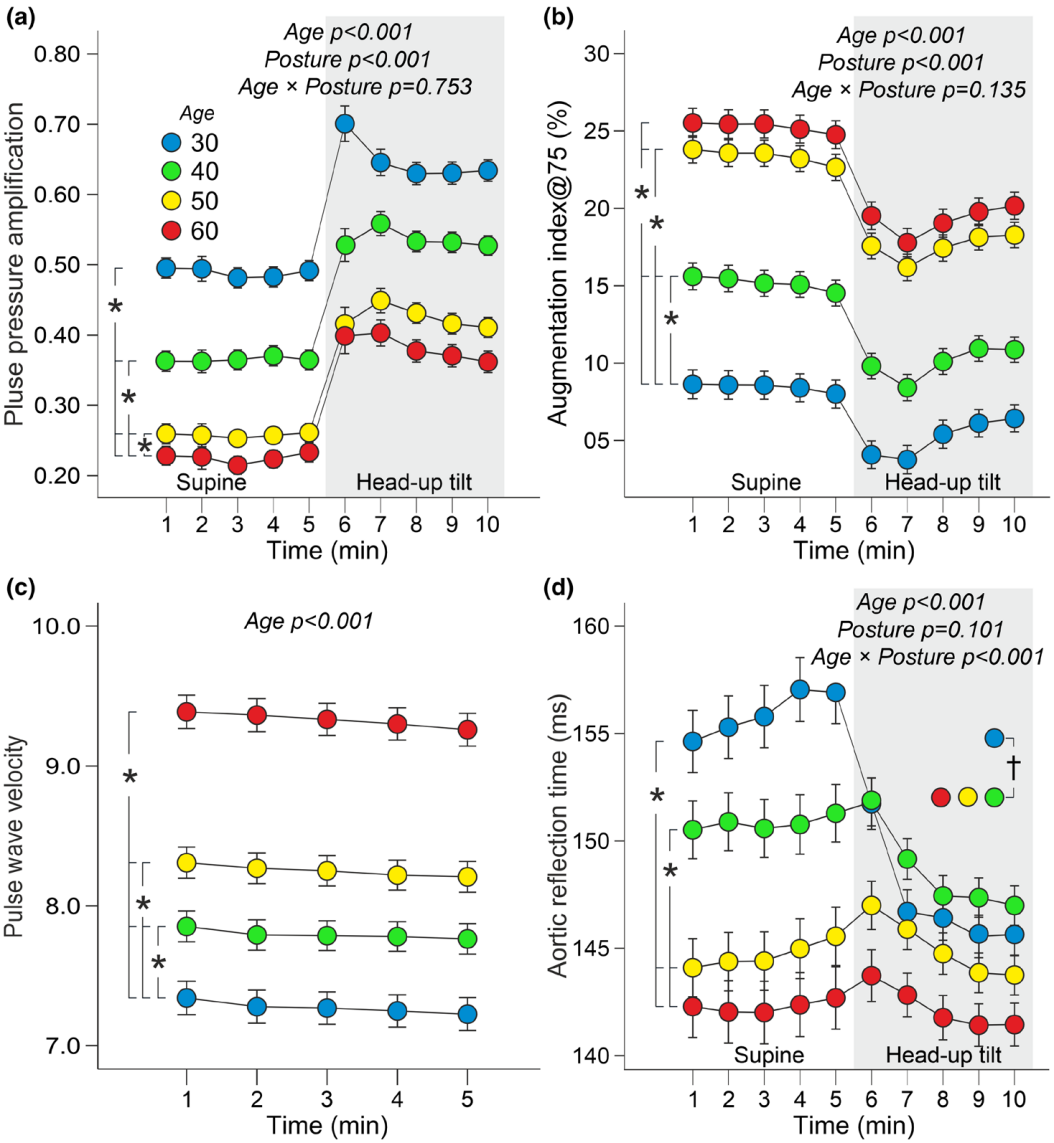
Line graphs illustrate pulse pressure amplification (a), augmentation index adjusted to heart rate 75/min (b), pulse wave velocity (c), and aortic reflection time (d) during supine position and passive head‐up tilt in different age groups. Statistical analyses using generalized estimating equations, adjustments for sex, body mass index, plasma lipids, and cystatin C, and mean aortic pressure in the case of pulse wave velocity; data are presented as mean and standard error of the mean to illustrate the precision of group means; *p < 0.05 between age groups, †p < 0.05 for the interaction between age and posture.

Pulse pressure amplification decreased with age (Figure 3a), whereas PWV increased with age (Figure 3c). AIx@75 was lowest in age group 30 and highest in age groups 50 and 60 (Figure 3b). Aortic reflection time was longest in age group 30 and shortest in age groups 50 and 60 (Figure 3d).

During the HUT, the increase in pulse pressure amplification and the decrease in AIx@75 were similar across all age groups (Figure 3a,b). However, the decrease in aortic reflection time during HUT was most pronounced in the 30‐year age group (Figure 3d). The decrease in aortic reflection time showed a moderate correlation with HUT‐induced shortening of cardiac ejection duration (*r*_P_ = 0.361, *p* < 0.001), and low correlations with PWV (*r*_P_ = 0.191, *p* < 0.001) and HUT‐induced increase in SVR (*r*_P_ = 0.187, *p* < 0.001).

**FIGURE 4:**
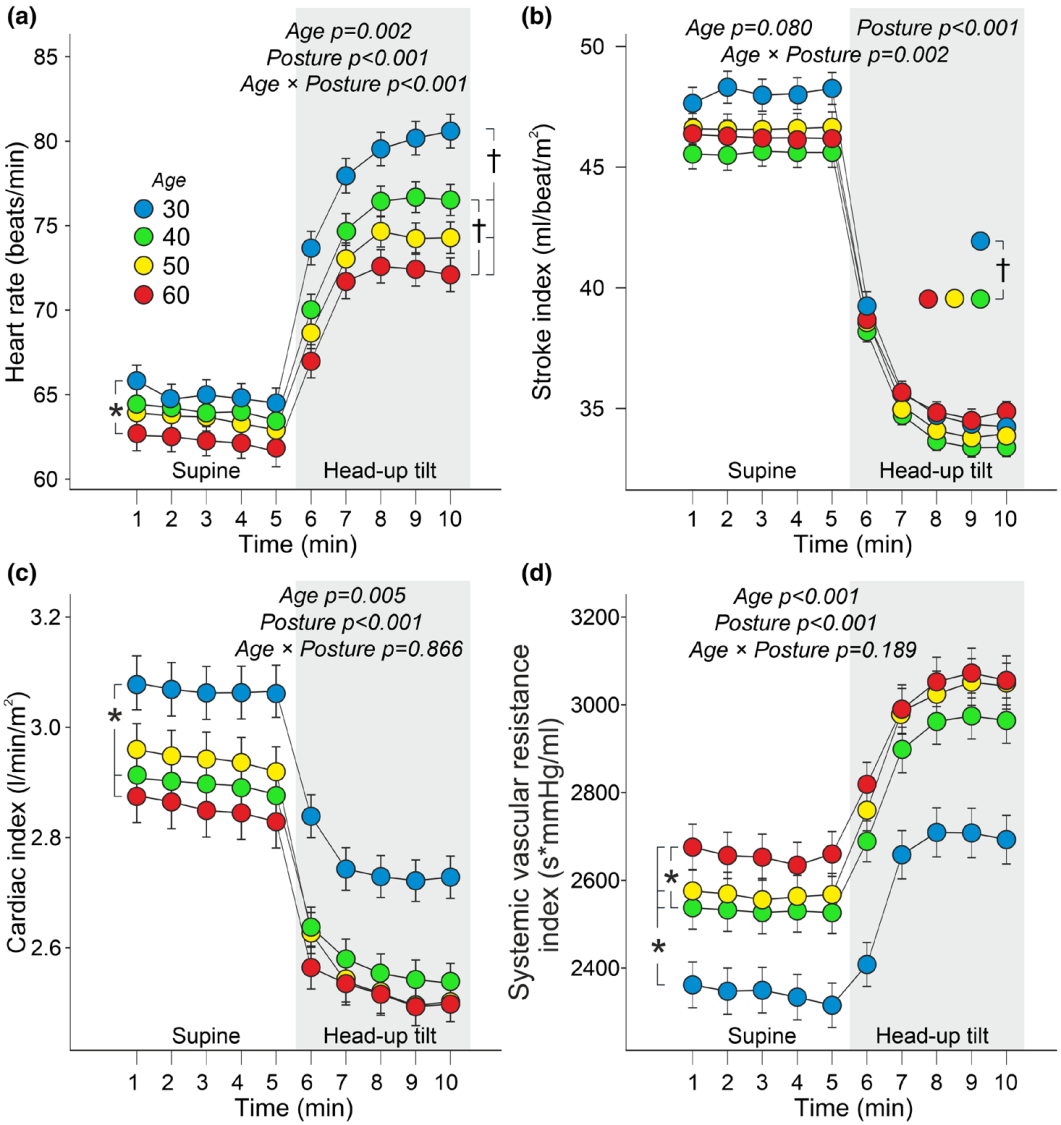
Line graphs illustrate heart rate (a), stroke index (b), cardiac index (c), and systemic vascular resistance index (d) during supine position and passive head‐up tilt in different age groups. Statistical analyses using generalized estimating equations, adjustments for sex, body mass index, plasma lipids, and cystatin C; data are presented as mean and standard error of the mean to illustrate the precision of group means; *p < 0.05 between age groups, †*p* < 0.05 for the interaction between age and posture.

In age group 30, heart rate was higher than in age group 60, and cardiac index was higher than in age groups 50 and 60 (Figure 4a,c). Stroke index did not differ significantly between the study groups (Figure 4b). Supine ejection duration was similar in all study groups (Table 1).

During HUT, heart rate increased most in age group 30 and more in age group 40 than in age group 60 (Figure 4a). Ejection duration during HUT was shortest in age group 30 and also shorter in age group 40 than in age group 60. Upright shortening of ejection duration was most marked in age group 30 (Table 1). The decrease in stroke index during HUT was most pronounced in age group 30 (Figure 4b). SVR index was lowest in age group 30 and lower in age group 40 compared to age group 60 (Figure 4d). The decrease in cardiac index and the increase in SVR index during HUT were consistent across all study groups (Figure 4c,d).

### 4.3 Heart rate variability

**FIGURE 5:**
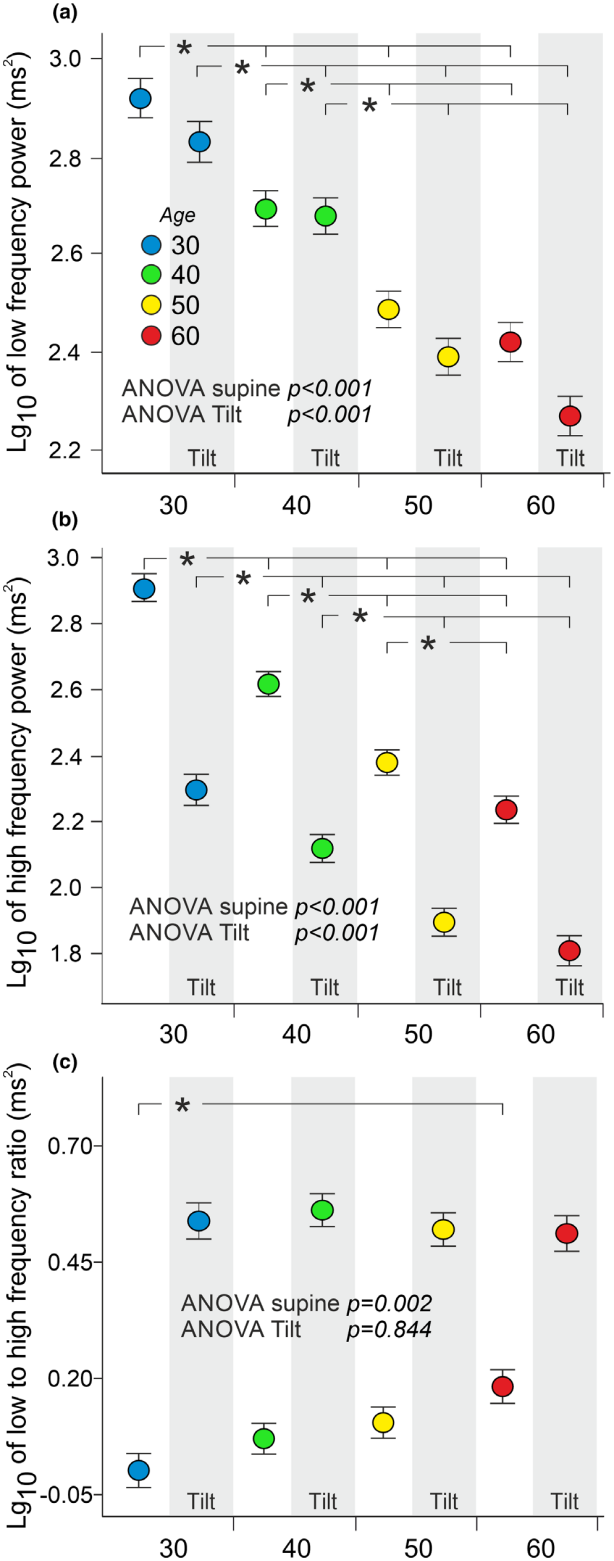
Heart rate variability (HRV) power in the low frequency (a) and high frequency (b) range, and low to high frequency ratio of HRV (c) during supine position and passive head‐up tilt in the different age groups. White background indicates supine, and gray indicates head‐up tilt; statistical analyses adjusted for sex, body mass index, plasma lipids, cystatin C, and mean supine and upright RR intervals; data are presented as mean and standard error of the mean to illustrate the precision of group means; *p < 0.05.

HRV in the LF power was highest in individuals in their 30s, followed by those in their 40s, compared to the two older age groups in both supine and upright positions. Regardless of posture, no significant difference in LF power was observed between individuals in their 50s and 60s (Figure 5a).

Supine HF power of HRV showed an age‐related decrease, with differences observed between all study groups. During the HUT, HF power of HRV decreased in all groups, with significant differences between all other study groups except for those in age groups 50 and 60 (Figure 5b).

The supine LF/HF ratio of HRV, which is considered to reflect sympathovagal balance in heart rate modulation (Task force of the European Society of Cardiology the north American Society of Pacing Electrophysiology, 1996), was higher in age group 60 compared to age group 30. However, during the HUT, the LF/HF ratio was similar across all study groups (Figure 5c).

## 5 DISCUSSION

To our knowledge, this is the largest HUT study to date (*n* = 522) focusing on age‐related hemodynamic changes in individuals without cardiovascular diseases or medications with direct cardiovascular effects. Key findings show that with aging, aortic systolic and diastolic BP, pulse pressure, PWV, wave reflection, and SVR increase, while heart rate, pulse pressure amplification, and aortic reflection time decrease. During HUT, aging was linked to more pronounced reductions in systolic BP and pulse pressure, along with a lower heart rate increase and less shortening of aortic reflection time. Aging also had a notable impact on HRV, both at rest and during HUT, highlighting the significant influence of age on autonomic cardiovascular responses. However, the attenuated heart rate response to upright posture in older age groups was not associated with a lower balance of sympathovagal modulation of heart rate.

The elevations in the systolic and diastolic BP observed between the age groups of 30 to 60 years in our study align with a gradual rise in systemic vascular resistance associated with aging (Franklin et al., 1997). High cardiac output in young adults, as observed in the present study, influences BP dynamics, leading to a reduced difference between systolic and diastolic BP due to the hyperkinetic circulation, where elevated cardiac output and relatively low SVR dominate the hemodynamic pattern (Li et al., 2023; Lund‐Johansen, 1991). In contrast, in older adults, SVR and arterial stiffness play a more significant role, consistent with our findings (Li et al., 2023; Lund‐Johansen, 1991). These findings align with the Framingham study, which documented an age‐related increase in systolic and diastolic BP among individuals aged 30 to 49 years, likely due to heightened peripheral vascular resistance (Franklin et al., 1997).

The increase in systolic and diastolic BP with increasing age in this study suggests three distinct hemodynamic phases. Before the age of 50, the progressive elevation in systolic and diastolic BP primarily reflects increased SVR. During the 50s, systolic BP increases but the rate of increase in diastolic BP slows down and eventually stabilizes (Franklin et al., 1997; Messerli et al., 1983). Concurrently, pulse pressure rises more steeply, indicating a combined effect of increased SVR and arterial stiffness. Beyond the fifth decade, a sharp rise in pulse pressure and continued increases in systolic BP, coupled with a stabilization in diastolic BP, highlight the growing impact of large artery stiffness (O'Rourke & Hashimoto, 2007). These changes occur alongside reductions in resting cardiac output and heart rate in elderly individuals, as also observed in the present study (Franklin et al., 1997; Messerli et al., 1983).

The continued rise in systolic BP in older individuals is predominantly attributed to structural changes in large arteries, such as elastin fragmentation, collagen deposition, and medial calcification. These alterations reduce compliance and increase stiffness, driving the late‐life rise in pulse pressure (Laurent et al., 2006; Laurent & Boutouyrie, 2020; Singam et al., 2019). It should be noted that augmentation index and PWV differed significantly already in younger age groups, with PWV showing an exponential increase in subjects aged 60 years. Pulse pressure amplification progressively decreases with age (Wilkinson et al., 2001), as observed in the present study. This decline is primarily due to increased arterial stiffness and enhanced wave reflection, which contribute to higher central pulse pressure and reduced peripheral amplification (Hashimoto & Ito, 2010; Wilkinson et al., 2001). Reduced arterial compliance with aging has been associated with impaired hemodynamic regulation, including a higher incidence of orthostatic hypotension (Mattace‐Raso et al., 2006). This age‐related decline in arterial compliance contributes to diminished baroreflex sensitivity, a critical mechanism for maintaining BP stability during postural changes (Laitinen et al., 2004). Reduced carotid artery compliance, a key determinant of cardiovagal baroreflex sensitivity, has been identified as a major factor driving this decline (Monahan et al., 2001). Thus, structural changes in large arterial walls, such as increased stiffness, exacerbate the loss of baroreflex sensitivity (Mattace‐Raso et al., 2006; Okada et al., 2012).

The shorter aortic reflection time observed in elderly subjects likely results from increased large artery stiffness and elevated SVR, which raise PWV and cause earlier wave reflection arrivals without a distal shift in reflection sites (Phan et al., 2016). A novel finding was that only the youngest age group showed a significant shortening of aortic reflection time in response to upright posture, a pattern lost with increasing age. More compliant arteries in younger individuals likely allow greater arterial dynamic changes during postural shifts (Davis et al., 2011; Tahvanainen, Leskinen, et al., 2009). In contrast, increased arterial stiffness in older individuals reduces the ability to adapt quickly to posture changes (Tahvanainen, Leskinen, et al., 2009). Younger participants also exhibited more pronounced increases in heart rate and decreases in ejection duration and stroke volume upon standing, likely contributing to the shortening of aortic reflection time (van den Bogaard et al., 2011). Notably, the decrease in aortic reflection time correlated most strongly with HUT‐induced shortening of ejection duration. Overall, multiple factors likely contributed to the lack of changes in aortic reflection time in older age groups.

While circulating catecholamine levels have been observed to rise with age—likely due to reduced neurotransmitter reuptake and diminished beta‐adrenergic receptor density (Esler et al., 1995; Fleg et al., 1985; Singam et al., 2019)—this compensatory mechanism may be insufficient to address the hemodynamic challenges associated with aging. In a previous study, 16 elderly men exhibited BP responses to HUT comparable to those of 13 younger males but displayed elevated resting norepinephrine levels and an exaggerated norepinephrine response to postural changes (Geelen et al., 2002). Altogether, the decline in baroreflex sensitivity with age may be more closely associated with increased arterial stiffness than with dysfunction of the efferent sympathetic or parasympathetic pathways (O'Mahony et al., 1979).

The response to HUT serves as a functional test for age‐related changes in cardiovascular autonomic regulation. Among 63 participants, divided into three age groups of 18–25 individuals each, younger individuals exhibited greater increases in heart rate and LF:HF power ratio during HUT, which were interpreted as indicators of effective autonomic regulation (Laitinen et al., 2004). In contrast, older adults demonstrated lower heart rate and LF:HF power during HUT, accompanied by a greater increase in peripheral arterial resistance. These findings were considered to reflect augmented vasoactive mechanisms with advancing age (Laitinen et al., 2004).

In our analysis, we observed a consistent age‐related decline in LF and HF power across quartiles, regardless of body position. Diminished supine and upright LF and HF power have been reported previously in several studies on aging (Bouquin et al., 2024; Laitinen et al., 2004; Schwartz et al., 1991; Strait & Lakatta, 2012; Yo et al., 1994). Analyses of electrocardiogram recordings have also demonstrated that both the HF and LF components of HRV decline with advancing age (Tsuji et al., 1996; Yeragani et al., 1997).

Consistent with prior research (Laitinen et al., 2004; Schwartz et al., 1991; Yo et al., 1994), we observed a significant reduction in HF power during HUT across all age groups, indicating that parasympathetic deactivation of cardiac control is a major adaptive mechanism during upright posture (Bouquin et al., 2024). Subsequently, the increase in the LF:HF ratio during HUT was primarily driven by a marked reduction in HF power, emphasizing that the rise in the LF:HF ratio is largely influenced by parasympathetic withdrawal. Altogether, the LF:HF ratio is a complex variable and cannot be interpreted solely as a marker of sympathetic overactivity (Schwartz et al., 1991; Yo et al., 1994).

In the present study, the LF:HF ratio during HUT did not significantly change across age groups. Thus, the age‐related reduction in the heart rate response to upright posture cannot be attributed solely to an imbalance in sympathovagal modulation. Lower upright heart rate could reflect the previously reported decrease in the beta‐adrenergic receptor density of the heart (Esler et al., 1995; Singam et al., 2019). Previous studies have also found that the upright LF:HF power ratio remains unaffected by age (Schwartz et al., 1991; Yo et al., 1994).

The present findings revealed that the oldest age quartile exhibited a higher supine LF:HF ratio compared to the youngest quartile. Previous studies have reported a higher supine LF:HF ratio of HRV in middle‐aged individuals (40–59 years) compared to younger adults (23–39 years). Additionally, an analysis of 24‐h electrocardiograms among 33 healthy subjects demonstrated a positive correlation between age and the LF:HF ratio, suggesting an age‐related increase in the LF:HF ratio (Yeragani et al., 1997). The elevated supine LF:HF ratio of HRV in elderly individuals, despite reduced cardiac output, may reflect the combined effects of decreased ventricular compliance, increased large arterial stiffness, and a compensatory rise in SVR to maintain BP (Strait & Lakatta, 2012; Yeragani et al., 1997).

The observed age‐related changes in central BP, PWV, wave reflections, SVR, and HRV during passive head‐up tilt suggest that these non‐invasive measures may serve as early indicators of vascular and autonomic aging. Incorporating functional hemodynamic and autonomic assessments, such as those conducted during orthostatic challenges, could improve the early detection of subclinical cardiovascular dysfunction, especially in individuals without overt disease (Dorogovtsev et al., 2024; Laurent et al., 2006, 2019; Parati et al., 2013). Establishing age‐specific normative ranges for these responses may help differentiate physiological aging from early pathological changes and support personalized risk stratification in clinical settings (Mitchell, 2008).

The current study has limitations, and the results should be interpreted with caution. Elderly subjects not taking antihypertensive medications were included, which may introduce selection bias, as those on such medications were excluded. Notably, a 2017 survey reported that the prevalence of hypertension among Finnish individuals over the age of 50 exceeded 60% in men and 50% in women (Koponen et al., 2019). While most participants were not using medications with direct cardiovascular effects, the potential influence of other medications on the results cannot be excluded. Although the methods used in this study have been validated against invasive measurements, three‐dimensional ultrasound, and PWV recordings (Kööbi et al., 1997; Koskela et al., 2013; Wilenius et al., 2016), the non‐invasive assessments of stroke volume and cardiac output rely on the mathematical analysis of the bioimpedance signal, which simplifies the underlying physiology (Kööbi et al., 1997). Similarly, central BP was mathematically derived from the radial artery tonometric signal (Chen et al., 1997). The recordings lasted 10 min, providing a relatively short window to assess hemodynamics. However, compared to single BP and heart rate measurements, this study's analysis was based on over 600 cardiac cycles per participant. Consequently, the average values for all hemodynamic variables during each minute represented a substantial number of individual data points. Despite some criticisms regarding the reliability of tonometric BP recordings (Nelesen & Dimsdale, 2002; Weiss et al., 1996), recent findings from our group demonstrate that these BP values align well with ambulatory daytime BP measurements in a cohort of 410 participants (Värri et al., 2023). Finally, the cross‐sectional nature of this study limits causal inferences, and the findings should be validated in longitudinal studies.

In conclusion, aging was associated with elevated BP and reduced HRV. The age‐related increases in BP and pulse pressure, along with reductions in pulse pressure amplification and aortic reflection time, were likely attributed to increased large arterial stiffness and SVR. While the heart rate response to upright posture diminished with advancing age, this change was not associated with an imbalance in sympathovagal modulation of HRV but may reflect the previously reported reduction in cardiac beta‐adrenergic receptor density (Esler et al., 1995; Singam et al., 2019).

## AUTHOR CONTRIBUTIONS

Conceptualization, data curation, formal analysis, funding acquisition, investigation: all authors. Methodology: JKK, EP, IHP. Project administration: IHP. Resources: MKC, JKK, LS, MH, JTM, PIN, IHP. Supervision: JTM, PIN, IHP. Validation: HB, JKK, KH, IHP. Visualization: MKC, IHP. Writing—original draft preparation: MKC, IHP. Writing review and editing: all authors.

## FUNDING INFORMATION

This work was supported by Finnish Foundation for Cardiovascular Research (IP, MKC), Tampere Tuberculosis Foundation (MKC), Aarne Koskelo Foundation (MKC), Sigrid Jusélius Foundation (IP), Competitive State Research Financing of the Expert Responsibility Area of Tampere University Hospital (IP, 9AB057 and 9 AC076), Finnish Medical Foundation (JKK, MKC), Päivikki and Sakari Sohlberg Foundation (JKK, MKC), and Pirkanmaa Regional Fund of the Finnish Cultural Foundation (JKK, MKC).

## CONFLICTS OF INTEREST

The authors declare no conflicts of interest.

## Supporting information


Data S1.

## Data Availability

Analyses and generated datasets that support the current study are not available publicly. The datasets are available from the corresponding author on reasonable request.
