# Supplementary material for: Impact of aging on cardiovascular dynamics and heart rate variability during passive head‐up tilt
Source: Physiol Rep. 2025 Jul 20;13(14):e70477. doi: 10.14814/phy2.70477 (PMC12277527; doi:10.14814/phy2.70477)
Supplement: Supplementary file 1 — Data S1. [file PHY2-13-e70477-s001.pdf]

RESEARCH ARTICLE

RUNNING HEAD: AGING AND HEMODYNAMICS

Impact of aging on cardiovascular dynamics and heart rate variability during passive head-up tilt

Manoj Kumar Choudhary[1], Kati Holmström[1,2], Heidi Bouquin[1], Tuomas P. Saarinen[1], Jenni K. Koskela[1,3], Essi Pietilä[1], Lauri Suojanen[1,3], Jukka T. Mustonen[1,3], Pasi I. Nevalainen[3], and Ilkka H. Pörsti[1,3,4*]

[1]Faculty of Medicine and Health Technology, Tampere University, Tampere, Finland;

[2]Department of Internal Medicine, Hospital Nova, Jyväskylä, Finland;

[3]Department of Internal Medicine, Tampere University Hospital, Tampere, Finland;

[4]Finnish Cardiovascular Research Centre Tampere, Tampere University, Tampere, Finland.

Correspondence: *Ilkka Pörsti (ilkka.porsti@tuni.fi)

Supplemental Figure S1. Histograms show the distribution of supine systolic (A, C, E, G) and diastolic (B, D, F, H) blood pressure among the study participants (n = 522) stratified by age groups: 30s (A and B), 40s (C and D), 50s (E and F), and 60s (G and H).

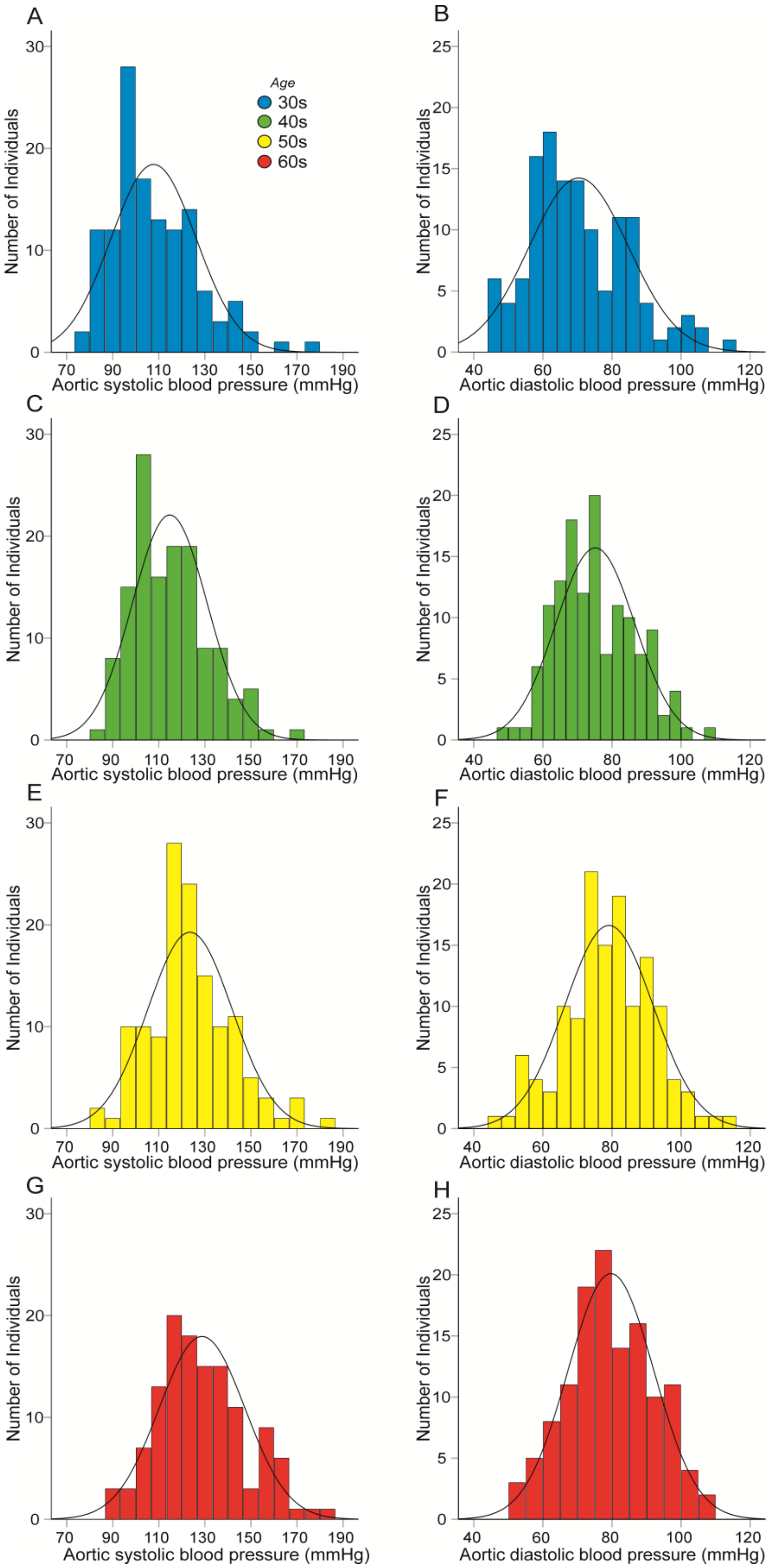

Supplemental Figure S2. Histograms show the distribution of supine heart rate among the study participants (n = 522), stratified by age groups: 30s (A), 40s (B), 50s (C), and 60s (D).

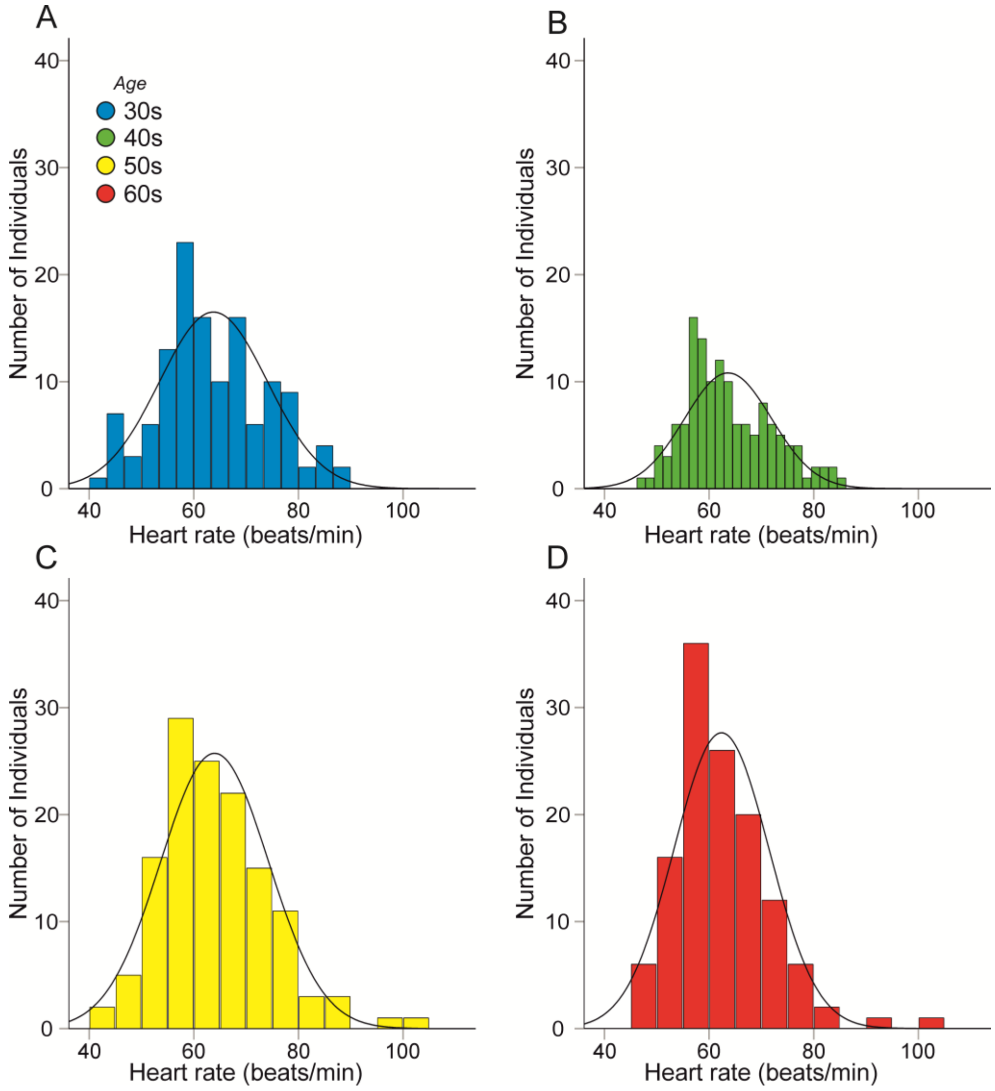

Supplemental Table. Medications and supplements among the study participants (n = 522) stratified by age groups.

**Medication / Supplement**	**Age (rounded to the nearest full decade)**				
	**30 (n=128)**	**40 (n=135)**	**50 (n=133)**	**60 (n=126)**	**p-value**
Acetylsalicylic acid (low dose)	1	0	1	4	0.346
Allopurinol	0	1	1	0	0.593
Antidepressant	3	6	13	10	0.322
Antihistamine	6	3	4	3	0.647
Anxiolytic	0	1	2	0	0.315
Acetylsalicylic acid (low dose)	1	0	1	4	0.346
Dietary supplement (vitamin, mineral)	18	27	23	27	0.437
Estrogen topically	0	1	4	1	0.118
Ezetimibe	0	0	0	1	0.369
Female hormone orally	21	16	19	18	0.771
Hypnotic	1	1	2	0	0.588
Inhaled beta2-mimetic	2	1	3	1	0.673
Inhaled glucocorticoid	4	4	4	3	0.985
Iron supplementation	0	1	1	0	0.593
Intra-uterine device, hormonal*	1	12	8	2	**0.018**
Magnesium supplement	0	1	0	1	0.570
Multivitamin supplement	2	4	4	1	0.524
Non-steroidal anti-inflammatory ager	0	0	4	1	0.222
Omega-3 supplement	0	0	2	3	0.130
Proton pump inhibitor	1	1	5	6	0.374
Statin**	1	0	8	4	**0.042**
Thyroxin	3	4	1	7	0.137
Warfarin	0	1	0	0	0.412

*More prevalent in age group 40 than in age group 30 (p=0.012),
**more prevalent in age group 50 than in age group 40 (p=0.024),
Chi-Square test with Bonferroni corrections.
